# Internal mammary artery perforator-based plug flap

**DOI:** 10.1016/j.ijscr.2024.110567

**Published:** 2024-11-09

**Authors:** Nello Pirozzi, Nicola Rocco, Maurizio Bruno Nava, Maíra Teixeira Dória, Camilla Victoria Weigert, Cícero de Andrade Urban

**Affiliations:** a Breast Unit, University of Naples Federico II, Naples, Italy; b Department of Advanced Biomedical Sciences, University of Naples Federico II, Naples, Italy; c Fondazione G.Re.T.A. (Group for Reconstructive and Therapeutic Advancements) ETS, Naples, Italy; d Hospital Nossa Senhora das Graças, Curitiba, Brazil

**Keywords:** Breast surgery, Oncoplastic surgery, Plastic surgery, Perforator flap, Case report

## Abstract

**Introduction:**

Various oncoplastic techniques have emerged over the years to preserve breast cosmesis and symmetry without compromising the principles of tumor excision. One of the newer techniques for breast volume replacement to achieve symmetry and cosmesis is the use of fascio-cutaneous pedicled chest wall perforator flaps or local perforator flaps (CWPF).

**Case presentation:**

We present a case of reconstruction with internal mammary artery perforator (IMAP)-based plug flap to fill the infero-medial defect caused by a tumor close to skin, with visible retraction.

**Clinical discussion:**

A 52 years old woman, with an extensive palpable mass (3 cm) in the lower medial quadrant of the right breast, the tumor was close to skin, with visible retraction. The patient has small and round breasts, without ptosis.

**Conclusion:**

In this situation and when there is skin that needs to be removed, reconstruction can be done with a pedicle flap skin paddle; the IMAP flap is an ideal donor site in these cases. It is a safe flap with good vascularization and offers a great cosmetic result.

## Highlights

• The IMAP flap is a good indication for patients with tumors close to the skin.
• The IMAP flap is a good indication for patients with small breasts without ptosis.
• Breast conserving therapy is equivalent to that of mastectomy in early-stage cancer.
• The IMAP flap is very useful for tumors in the middle quadrants of the breast.
• Oncoplastic involves a combination of anatomical knowledge and oncological principles.

## 1 Introduction

Breast-conserving therapy (BCT) is defined as a breast conserving wide local excision (WLE) of a mammary tumor combined with postoperative radiotherapy [1,2]. The overall survival rate of BCT is equivalent to that of mastectomy for women with early-stage breast cancer [2,3]. Some recent population-based studies suggest that long-term survival of BCS with radiation may even be superior to mastectomy [4], while preservation of the remaining breast is associated with better psychosocial and psychosexual rehabilitation [5]. For this reason, the immediate restoration of the mammary shape by using oncoplastic surgery techniques is fundamental. Still, BCT is associated with a risk of deformation of the breast, particularly in cases where more than 20 % of the mammary volume is excised [6]. The medial quadrants of the breast are especially prone to such a deformation [7,8],especially when the tumor is close to the skin.

Oncoplastic surgery is one of the most complex areas of surgery. It involves a perfect combination of technique and art. Cancer does not give the opportunity to improvise on oncological principles. Reconstruction, on the other hand, often requires creativity. And the surgeon, during surgery, needs to have the sensitivity to recreate the shape and the beauty, respecting the limits of the patient's anatomy and autonomy, without exceeding oncological limits.

All attempts should be made to minimize the risk of positive margins, which are difficult and sometimes impossible to reassess in a second surgery, and to reduce and prevent complications that may delay adjuvant treatments. The ideal location of a tumor is within the resection area of the mammaplasty. When the tumor is close to the skin and outside this area, the oncoplastic (OP) procedure may be more complex and require combined techniques, whose results are not always satisfactory [9].

There are certain clinical situations in which the local involvement makes it necessary to use anatomical resources to close surgical wounds, or to avoid complications, such as necrosis and skin loss. Several techniques have emerged over the years for the preserve breast cosmesis without compromising the principles of tumor excision. One of these more recent technique for breast volume replacement after oncological surgery is the use of chest wall fascio-cutaneous pedicled local perforator flaps (CWPF) [10,11]. These flaps allow surgeons to close defects with less morbidity compared to muscle flaps, and also provide the opportunity to perform conservative surgery in cases when it is necessary to resect a large volume or a piece of skin [12].

It is important to consider the vascular anatomy of the anterior chest wall, which is a complex interconnected system: the perforating artery trunks and the subdermal plexus. The perforating arteries are responsible for the origin of the blood supply. Knowledge of its location and distribution is the most important element for the success of a local flap [12].

We describe the use of a round paddle of skin located in the internal mammary artery inferior branch (IMAP) to replace the defect left by the removal of a tumor very close to the skin, with visible retraction, which allowed us to perform a breast-conserving surgery in a patient with advanced local tumor and achieve significant cosmetic improvement in the reconstructed breast contour.

## 2 Case report

### 2.1 Clinical assessment

**Fig. 1:**
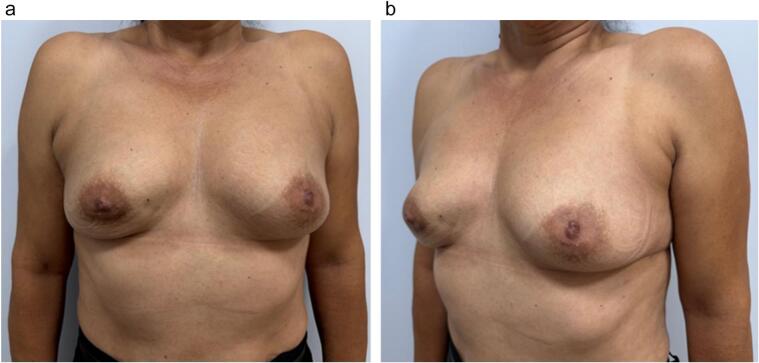
a,b: The patient has small and round breasts, without ptosis. The tumor was close to skin, with visible retraction.

A 52 years old woman, with an extensive palpable mass (3 cm) in the lower medial quadrant of the right breast. Pathology confirmed a lobular invasive carcinoma of the breast, luminal B. She is a smoking patient without other co-morbidities. No family history of cancer. The patient has small and round breasts, without ptosis. The tumor was close to skin, with visible retraction (Fig. 1a, b).

**Fig. 2:**
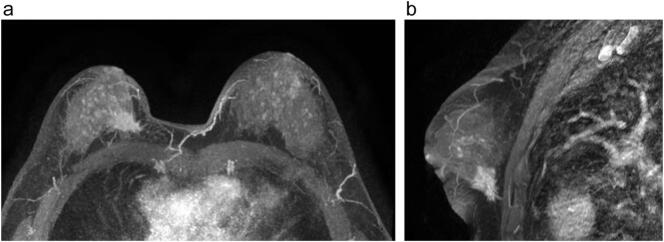
a,b: Magnetic resonance of the breast showing the mass in the lower medial quadrant of the right breast.

The magnetic resonance of the breast shows an irregular solid nodule, measuring 2.9 cm, with spiculated margins, in the lower medial quadrant of the right breast, and 3.4 cm from the nipple (Fig. 2).

The patient was informed about the different options and, in agreement with the multidisciplinary team, was then consented to IMAP flap reconstruction and was informed that if any complications occurred, the reconstruction would have been converted into a traditional one.

The work has been reported in line with the SCARE criteria [13].

### 2.2 Preoperative marking

**Fig. 3:**
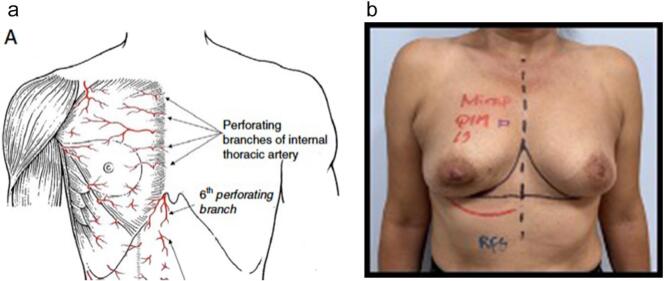
a: Anatomy of perforating branches of the anterior thorax wall showing the medial brach of the internal mammary artery (taken from Acea Nebril, B. et al. (2016) ‘Rotational flaps in oncologic breast surgery. anatomical and technical considerations’, *Cirugía Española (English Edition)*, 94(7), pp. 372–37). b: Preoperative planning for the plug flap technique using the inferior branch of the internal mammary artery as its base.

The patient was marked the day before surgery. The size of the flap was planned taking into account the inframammary fold, the location and size of the tumor and the territory of the inferior branch of the internal mammary artery (Fig. 3).

### 2.3 Surgical technique

**Fig. 4:**
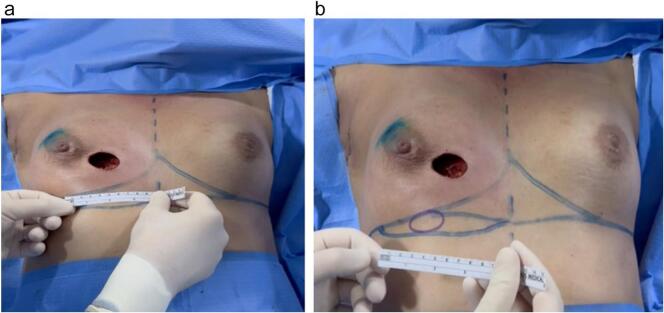
a,b: Skin defect measuring 3.5 cm and internally measuring approximately 5 cm in diameter. Intraoperative assessment of the rotation arc and planning of the plug flap area.

**Fig. 5:**
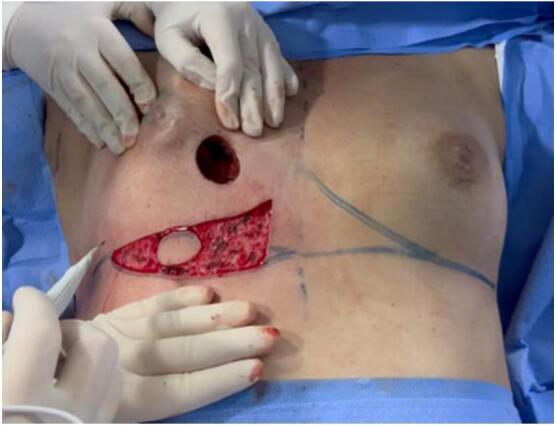
Delimitation of the skin flap and de-epithelialization.

**Fig. 6:**
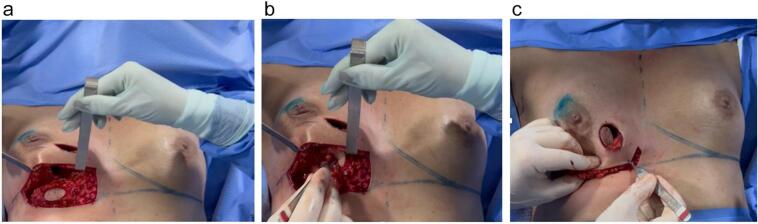
a,b,c: Preparation and rotation of the plug flap based on the inferior branch of the internal mammary artery.

The wide local excision was done with the portion of skin close to the tumor. Then the defect was measured and the paddle of skin for the plug flap was drawn at the exact distance of the rotation (Fig. 4). The IMAP flap was de-epithelialized preserving the paddle of skin to reconstruct the defect (Fig. 5).The flap was prepared and rotated to the breast defect (Fig. 6).

### 2.4 Post-operative management and results

**Fig. 7:**
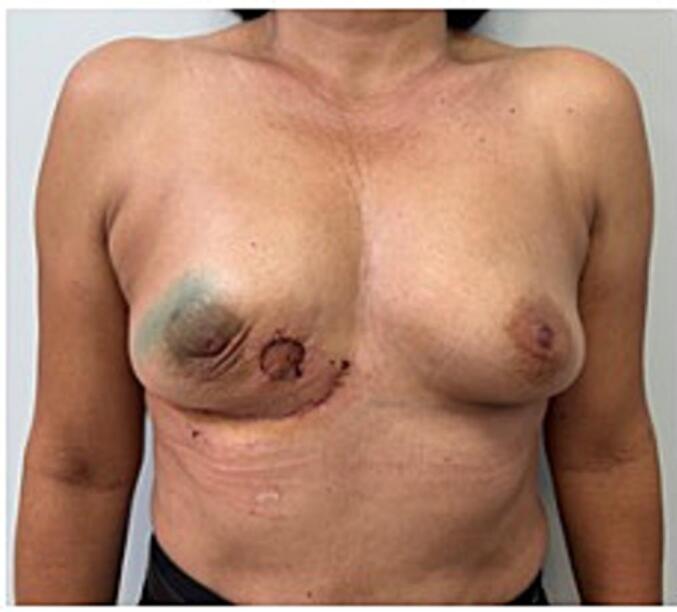
Result one week after surgery.

The patient was discharged the next day and scheduled to be seen at postoperative day 6. She was good, without any complications and the overall cosmetic results were extremely satisfactory for the patient and surgeons alike. The pathological analysis confirmed the initial findings with tumor-free margins (the smallest being 5 mm) and node negative (Fig. 7).

## 3 Discussion

It's important for breast surgeons have standardized oncoplastic training, as breast preservation offers greater aesthetic satisfaction and well-being to patients. There are tumors that are very close to skin or located in places that are more difficult to obtain a good cosmetic result. Local rotational flaps for breast conserving surgery are a group of surgical procedures that can be used in these situations, with lower morbidity than distant flaps, allowing adjuvant treatments to be initiated and in some case avoiding mastectomies with improved survivorship (satisfaction, function, and health-related quality of life) [14].

Cases with tumors in the middle quadrants of the breast are especially prone to deformities, particularly when more than 20 % of the mammary volume is excised [6]. In this situation and when there is skin that needs to be removed, reconstruction can be done with a pedicle flap skin paddle; the IMAP flap is an ideal donor site in these cases. It is a safe flap with good vascularization and offers a great cosmetic result.

It is essential to know the vascular anatomy of the perforating arteries to choose the appropriate flap. To our knowledge, this internal mammary artery inferior branch-based plug flap has not been described to correct breast skin defects and it is a good indication for patients with tumors close to the skin and with small breasts without ptosis.

## Consent

Written informed consent was obtained from the patient for publication and any accompanying images. A copy of the written consent is available for review by the Editor-in-Chief of this journal on request.

## Ethical approval

Ethics approval is not required for case reports, as they are deemed not constitute research at our Institution.

## Funding

None.

## Author contribution

Nello Pirozzi, Cicero Urban and Nicola Rocco created the concept of the case report.

Maurizio Bruno Nava, Maíra Teixeira Dória, Camilla Victoria Weigert are contributors.

## Guarantor

Nello Pirozzi.

Nicola Rocco.

## Research registration number

None.

## Conflict of interest statement

None declared.

## Appendix A Supplementary data

Supplementary Video IMAP Flap. Supplementary Video
